# Complete Genome Sequence of *Gardnerella vaginalis* Strain JCM 11026^T^, Isolated from Vaginal Tracts of Women

**DOI:** 10.1128/genomeA.00286-15

**Published:** 2015-04-09

**Authors:** Kenshiro Oshima, Shin Hisamatsu, Hidehiro Toh, Akiyo Nakano, Misa Kiuchi, Hiromi Kuroyanagi, Hidetoshi Morita, Masahira Hattori

**Affiliations:** a Graduate School of Frontier Sciences, University of Tokyo, Kashiwa, Japan; b School of Life and Environmental Science, Azabu University, Sagamihara, Japan; c Medical Institute of Bioregulation, Kyushu University, Fukuoka, Japan; d Department of Microbiology and Immunology, Teikyo University School of Medicine, Tokyo, Japan; e School of Veterinary Medicine, Azabu University, Sagamihara, Japan

## Abstract

*Gardnerella vaginalis* strain JCM 11026^T^ was isolated from vaginal tracts of women. Here, we report the complete genome sequence of this organism.

## GENOME ANNOUNCEMENT

*Gardnerella vaginalis* commonly occurs in the vaginal microbiota of healthy individuals and is classified under the family *Bifidobacteriaceae* (1). This species is most frequently isolated from women with bacterial vaginosis and often produces a cholesterol-dependent cytolysin and vaginolysin (2). *G. vaginalis* urogenital biofilm also increases in inflammatory bowel disease, and this observation suggests an epithelial barrier dysfunction of the genital tract (3).

*G. vaginalis* strain JCM 11026^T^ (ATCC 14018^T^) was isolated from vaginal tracts of women. We determined the complete genome sequence of *G. vaginalis* JCM 11026^T^ using a whole-genome shotgun strategy with Sanger sequencing (ABI 3730xl sequencers). We constructed small-insert (2-kb) and large-insert (10-kb) genomic DNA libraries and generated 26,880 sequence reads (11.8-fold coverage) for *G. vaginalis* JCM 11026^T^ from both ends of the genomic clones. Data were assembled with the Phred-Phrap-Consed program. Gap closing and resequencing of low-quality regions were conducted by Sanger sequencing to obtain the high-quality finished sequence. The overall accuracy of the finished sequence was estimated to have an error rate of <1 per 10,000 bases (Phrap score of ≥40). An initial set of predicted protein-coding genes was identified using Glimmer version 3.0 (4). Genes consisting of <120 bp and those containing overlaps were eliminated. The tRNA genes were predicted by tRNAscan-SE (5), and the rRNA genes were detected by a BLASTn search using known *Gardnerella* rRNA sequences as queries.

The genome sequence of *G. vaginalis* JCM 11026^T^ consists of a circular chromosome of 1,667,406 bp with no plasmid. The genome size is smaller than those of the *Bifidobacterium* species, whose genomes range in size from 1.9 to 2.8 Mbp (6). The genome of JCM 11026^T^ has a GC content of 41.4%, which is much lower than those (56% to 63%) of bifidobacterial genomes. JCM 11026^T^ contained a clustered regularly interspaced short palindromic repeats (CRISPR) (7, 8) region (697, 302 to 699, 159), and eight CRISPR-associated genes (GAVG_0509 to GAVG_0516) were encoded upstream of the CRISPR region. The chromosome contains 1,276 predicted protein-coding genes. We compared the genome of JCM 11026^T^ with publicly available complete genome sequences of *G. vaginalis* strains (ATCC 14019 and strain 409-05) (9). JCM 11026^T^ shared 1,247 and 1,005 protein-coding genes with ATCC 14019 and strain 409-05, respectively, and 1,003 protein-coding genes were common to the three strains. JCM 11026^T^ contained genes for both type I and II pili (9) and, in addition, encoded a cell surface protein cluster (GAVG_0453 to GAVG_0462), which is absent in strain 409-05. Whole-genome alignment of JCM 11026^T^ and ATCC 14019 showed their colinearity, and that of JCM 11026^T^ and strain 409-05 revealed a broken X-pattern, which is generated by symmetric chromosomal inversions around the origin of replication. The genome information of this species will be useful for further studies of its physiology, taxonomy, clinical aspects, and ecology.

### Nucleotide sequence accession number.

The sequence data for the genome described here have been deposited in DDBJ/GenBank/EMBL under the accession number AP012332.
